# Impact of establishing a level-1 trauma center for lower extremity trauma: a 4-year experience

**DOI:** 10.1186/s12873-022-00682-w

**Published:** 2022-07-07

**Authors:** Min Ji Kim, Kyung Min Yang, Hyung Min Hahn, Hyoseob Lim, Il Jae Lee

**Affiliations:** Department of Plastic and Reconstructive Surgery, Ajou University School of Medicine, 164, World cup-ro, Yeongtong-gu, Suwon, 16499 Korea

**Keywords:** Amputation, Limb salvage, Lower limb, Open fracture, Trauma center

## Abstract

**Purpose:**

A multidisciplinary approach is essential for trauma patients’ treatment, particularly for cases with open lower extremity fractures, which are considered major traumas requiring a comprehensive approach. Recently, the social demand for severe-trauma centers has increased. This study analyzed the clinical impact of establishing a trauma center for the treatment of open lower extremity fractures.

**Methods:**

A retrospective chart review was conducted for trauma patients admitted to our hospital. Patients were classified into two groups: before (January 2014–December 2015, 178 patients) and after establishment of a Level-1 trauma center (January 2017–December 2018, 125 patients). We included patients with open fracture below the knee level and Gustilo type II/III, but excluded those with life-threatening trauma that affected the treatment choice.

**Results:**

Total 273 patient were included in this study, initial infection was significantly more common and external fixator application significantly less in post-center establishment group. The time to emergency operation decreased significantly from 13.89 ± 17.48 to 11.65 ± 19.33 h post-center setup. By multivariate analysis, the decreased primary amputation and increased limb salvage was attributed to establishment of the trauma center.

**Conclusion:**

With the establishment of the Level-1 trauma center, limbs of patients with open lower extremity fractures could be salvaged, and the need for primary amputation was decreased. Early control of initial open wound infection and minimizing external fixator use allowed early soft tissue reconstruction. The existence of the center ensured a shorter interval to emergency operation and facilitated interdepartmental cooperation, which promoted active limb salvage and contributed to patients’ quality of life.

## Introduction

Trauma and its sequelae impose a socio-economic burden. Lower extremity trauma, which is a common traumatic accident, directly affects patients’ walking ability and thereby markedly influences the patients’ quality of life [1]. Therefore, appropriate interventions involving multidisciplinary cooperation should be initiated promptly to minimize traumatic damage. Through the systematic intervention of trauma surgeons, thoracic surgeons, orthopedic surgeons, neurosurgeons, and other surgeons, it is possible to save lives while minimizing complications. A structured treatment system involving each department affiliated with a trauma center is crucial for prompt treatment. In Korea, public attention to industrial accidents is increasing with industrialization and advances in technology. As the number of and degree of interest in industrial accidents increase, this issue is gaining in social and political importance.

In terms of quality of life, particularly as relates to the anatomy of the lower limbs, prompt and organized management is required. First, soft tissue damage requires treatment, because the lower limb has pliable skin, so that direct trauma easily causes skin defects, and fracture-related circular swelling results in skin necrosis. The wound is usually combined with bone and tendon exposure; thus, lower limb trauma with soft tissue defects is challenging for the reconstruction surgeon. In open fracture of the lower limb with extensive soft tissue defects, reconstruction surgery is vital, because such injuries can lead to amputation.

There are various methods of reconstruction of open fracture with soft tissue defects involving the lower limbs, for example, from a local flap to a cross-over flap and a free flap [2]. Among the various reconstructive options, the free flap has been most successful, possibly due to the recent advances in microsurgery. Many reports have demonstrated that free flap reconstruction for lower limb trauma is a successful strategy for limb salvage [3]. Through this surgical technique, lower limb salvage in such trauma cases has increased upto 18% [3–5]. For wounds needing reconstruction there remains no other alternative than amputation. As amputation severely reduced the patients’ quality of life, various attempts are made to salvage the limb.

However, microsurgical lower limb reconstruction requires establishment of a trauma center. To address this need, locoregional and social demands has led to the establishment of the largest regional Level-1 trauma center at our institution in 2006. Its cost-effectiveness and its important role in improving patients’ quality of life have been reported previously [6, 7]. These previous studies have focused on major mainstream departments, such as trauma surgery, orthopedic surgery, and thoracic surgery, in the trauma center [1]. However, successful limb salvage requires a multidisciplinary approach, in which each involved department plays an important role.

The purpose of this study was to analyze the clinical impact of establishing a trauma center on the treatment of open lower extremity fractures. Considering the various involved departments, we discuss the clinical changes and patterns of limb salvage and amputation in cases of open lower extremity fractures with soft tissue damage before and after establishment of the trauma center, and address the role of reconstructive surgeons in trauma centers.

## Patient and methods

### Study design

A retrospective study was conducted on patients with open fractures of the lower leg from January 2014 to December 2018. Patients were grouped according to their admission to our institution before or after establishment of the Level-1 trauma center. Since the center opened in 2016, this 1-year period was excluded from the statistical analysis due to the possible bias caused by the transition period. The inclusion criterion was an open fracture of the lower limb(s), below the knee, which required local soft tissue reconstruction or primary closure. Exclusion criteria were patient death, injury at other sites, a soft tissue defect above the knee, and amputation of the toe. The need for informed consent was waived due to the retrospective nature of the study.

### Level-1 trauma center establishment

In 2012, 15 trauma centers were established under the Korean policy to establish a national trauma system. Our institution was designated as a regional trauma center in 2013, and a complete trauma center was opened in March 2016 [8]. Our institution is thus an independent, nationally certified center that is categorized as a Level-I trauma facility by the Korean Surgical Society, with the capacity to treat and care for pediatric patients [9]. Surgeons able to treat trauma patients were employed in a timely manner. In addition to the medical staff that operate the trauma center, trauma coordinators, trauma registrars, trauma program managers (TPMs), and other relevant personnel were employed. All staff and equipment dedicated to treating trauma patients receive government financial support. In addition, the number of beds, the nurses employed, and equipment purchased were of world standard. The trauma center is equipped with three independent operating rooms that are used only for trauma patients. To improve performance, we applied a quality improvement program through the American College of Surgeons Committee on Trauma (ACSCOT) and World Health Organization (WHO) Trauma Quality Improvement Program (TQIP) guidelines, in accordance with the operating standards and guidelines for US level I trauma center [10].

At the trauma bay in our center, acute injury is initially managed by the attending trauma surgeon, who decides on the intervention and specialist care required in cases with lower limb trauma. A plastic surgeon can involved from the start, as there may be a need for open fracture or soft tissue reconstruction.

### Assessment

Medical records were reviewed to obtain patient demographic and comorbidity data. In wound assessment, the presence or absence of infection was evaluated. To assess the severity of open lower leg fractures, we evaluated the Gustilo‒Anderson Classification of each patient [11, 12]. In the case of bone loss, which is a major condition for amputation, bone loss of 5 cm or more was confirmed by radiological evaluation. Lower extremity contrast-enhance angio-computed tomography (CT) was initially performed to determine the degree of vessel injury, which was confirmed by angiography when vessel injury was suspected on CT. The main vessels of the lower limbs were divided into the anterior tibial artery, posterior tibial artery, and peroneal artery. Use of an external fixator was identified when an external fixator was inserted during the first surgery at the time of admission on our hospital, or when an external fixator was inserted at the first hospital visited prior to transfer to our institution. To define the general condition of the patient immediately after the injury, initial intubation, transfusion, and intensive care unit (ICU) admission were recorded.

**Fig. 1:**
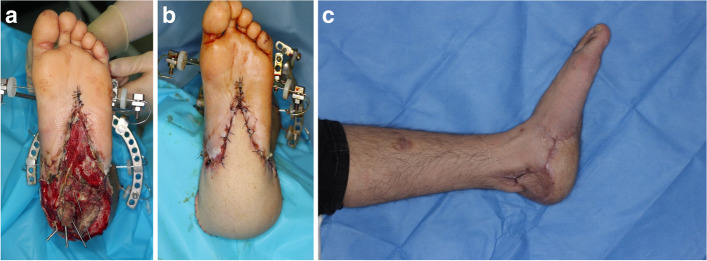
A case of open lower limb traumatic injury **A** A 20-years-old male who Injured by stepping on a landmine, was refered for reconstruction of soft tissue defect. Initially, calcaneal bone was exposed and open fracture was noted. Following debridement orthopedic surgeons performed external fixation with K-wire and Ilizarov system to preserve bony structure of injured foot. **B** After serial debridement with antibiotic therapy, The left anterolateral thigh musculocutaneous flap was elevated and then applied to the recipient site. In this patient, an end-to-end anastomosis was performed between the posterior tibial artery and the descending branch of lateral circumflex femoral artery. **C** The patient can escape from amputation and can achieved full ambulatory status at 6 months after the operation

**Fig. 2:**
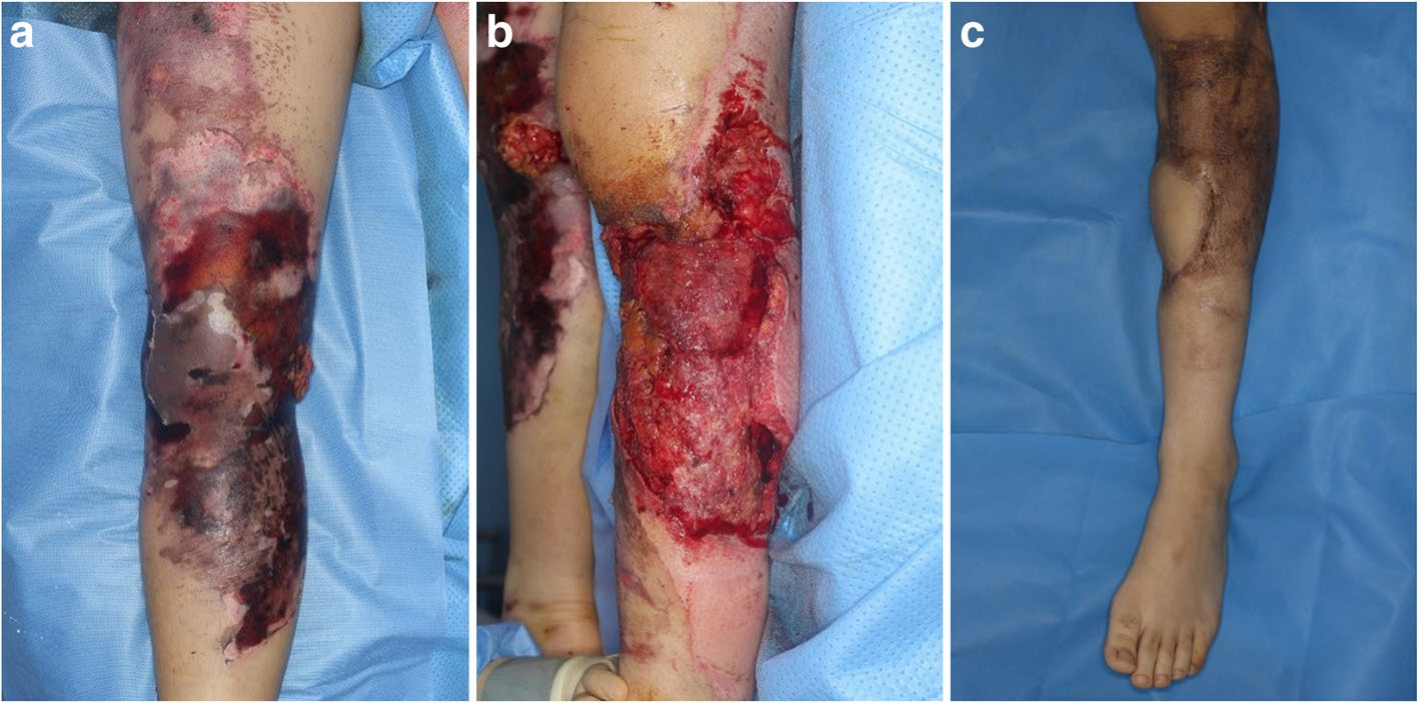
Representative case of open lower limb traumatic injury. **A** A 63-year-old man who was injured by mine injury. Tibiofibular fracture was noted and initial wound has no open wound. But the injuried site was necrotized gradullay then the knee joint exposured with tendon and muscle exposure. **B** Radical debridement was done and skin graft was proceeded to cover the defect. The exposured joint was covered with microsurgical free tissue transfer using an anterolateral thigh flap. **C** The patient can escape from amputation and can achieved full ambulatory status at 8 months after the operation

Resolution of soft tissue wounds without a requirement for major amputation was classified as limb salvage. Amputation was classified into primary and secondary amputation. Primary amputation was defined as a case in which amputation was performed as the first operation after being referred to the trauma center after an injury, and secondary amputation was defined as when amputation was decided on after the first operation. Patients with primary amputation and an external fixator were cases in which the initial care was external fixator insertion at another hospital followed by transfer to our institution, after which amputation was decided in the first operation at our institution (Fig. 1a, b, c). Complications were defined as the need for surgical intervention for infection or flap re-anastomosis or dehiscence after the final reconstructive surgery, e.g., free flap transfer and skin graft (Fig. 2a, b, c).

### Statistical analysis

Statistical analyses were performed with SPSS for Windows version 18.0 (SPSS Inc., Chicago, IL, USA). Data were presented as two standard deviations. Statistical significance was set at a *p*-value < 0.05. Pearson’s chi-square test was performed to evaluate qualitative data. Variables with a *p*-value < 0.05 were included in the multivariate analysis to identify factors contributing to the need for amputation.

## Results

The demographic details of the 273 patients with open lower limb fracture injuries included in the study are shown in Table 1. Slightly more than half of the patients were admitted before the center was established (group 1), while the rest were admitted after center establishment (group 2). The most common cause of injury was out of car traffic accident, followed by inside car traffic accident, and motorcycle accident. Emergency care included extended focused abdominal sonography for trauma (E-fast), transfusion, and intubation [13]. The location of the open fracture with the soft tissue defect was most commonly in the lower leg, followed by the foot.

**Table 1:** Patients demographics and baseline characteristics

Variables		n	%
Group	2014‒2015	145	53.1
	2017‒2018	128	46.9
Sex	Female	58	21.2
	Male	216	78.8
Age^a^		47.62	20.02
Height^a^		166.27	13.83
Weight^a^		65.96	14.41
BMI^a^		23.50	3.42
Cause of injury	Out car TA	84	30.9
	In car TA	53	19.5
	Fall down	45	16.5
	Contusion	36	13.2
	Motorcycle	54	19.9
DM		38	13.9
HTN		60	21.9
Others Comorbidities		35	12.9
Intubation		58	21.2
Transfusion		152	56.5
E-fast		85	31.0
ICU care		147	53.8
Combined injury	Orthopedic	271	44.9
	Neurosurgical	32	5.3
	Soft tissue injury	169	28.0
	Intra-organ injury	36	6.0
	Medical problem	68	11.3
	Others	27	4.5
Injury location	Foot	129	36.5
	Lower leg	182	51.6
	Thigh	42	11.9

^a^mean ± SD

*BMI* Body mass index, *ICU* Intensive care unit, *E-fast* Extended Focused Abdominal Sonography for Trauma, *TA* Traffic accident, *DM* Diabetes mellitus, *HTN* Hypertension

There was no significant difference in the proportion of the Gustilo‒Anderson type between the groups. The percentage of patients with existing infection among the lower limb trauma patients overall was significantly higher, and the percentage of patients with external fixator application at the initial step was significantly lower in group 2 than in group 1 (Table 2). The time to emergency operation decreased by an average of 2.24 h (from 13.89 ± 17.48 h before to 11.65 ± 19.33 h after center establishment, *p* < 0.031). Open wounds requiring soft tissue reconstruction, degloving injuries, and ischemic injuries requiring plastic surgery from the start increased from 46.1% before center establishment to 51.4% after center establishment. Among the various reconstructive options, use of a free flap increased by 50% from before to after center establishment (*p* < 0.001).

**Table 2:** Comparison of operative details in lower extremity trauma injury

		**Before center** **Group 1** **(n, %)**	**After center** **Group 2** **(n, %)**	**Total**	**χ**^**2**^** (p)**
**Gustilo‒Anderson type**	**l**	31 (21.4)	23 (18.0)	54 (19.8)	3.941 (0.558)
	**ll**	60 (40.7)	44 (34.4)	104 (37.7)	
	**lllA**	13 (9.0)	15 (11.7)	28 (10.3)	
	**lllB**	17 (11.7)	17 (13.3)	34 (12.5)	
	**lllC**	24 (16.6)	29 (22.7)	53 (19.4)	
**Existence of infection**		30 (20.7)	26 (23.5)	56 (20.6)	0.002 (0.005)
**Bony loss**	** < 5 cm**	132 (91.0)	113 (88.3)	245 (89.7)	0.560 (0.454)
	** ≥ 5 cm**	13 (9.0)	15 (11.7)	28 (10.3)	
**External fixator**		49 (33.8)	21 (16.5)	70 (25.7)	10.550 (0.001)
**Vessel injury**	**ATA**	3 (2.1)	6 (4.7)	9 (3.3)	5.307 (0.218)
	**PTA**	2 (1.4)	5 (3.9)	7 (2.6)	
	**Peroneal**	0 (0.0)	1 (0.8)	1 (0.4)	
	**Others**	3 (2.1)	5 (3.9)	8 (2.9)	
**Angioplasty**		1 (0.7)	5 (3.9)	6 (2.2)	3.272 (0.070)
**Time to emergency operation (hours, mean ± SD)**		13.89 ± 17.48	11.65 ± 19.33		0.031
**Plastic surgery department intervention**		59 (46.1)	74 (51.4)	133 (48.9)	11.760 (0.003)
**Reconstructive option**	**Skin graft**	17 (43.6)	20 (40.0)	37 (41.6)	19.363 (< 0.001)
	**Local flap**	6 (15.4)	2 (4.0)	8 (9.0)	
	**Free flap**	5 (12.8)	25 (50.0)	30 (33.7)	
	**Debridement**	11 (28.2)	3 (6.0)	14 (15.7)	
**Complications**		15 (10.3)	10 (7.8)	25 (9.2)	0.524 (0.469)
**Patient survival**		141 (97.2)	126 (98.4)	267 (97.8)	0.453 (0.501)

*ATA* Anterior tibial artery, *PTA* Posterior tibial artery

Chi-square analysis was performed to identify factors that contributed to either lower extremity amputation or salvage (Table 3). Intubation, Gustilo‒Anderson type, ICU care, more than 5 cm bone loss, and presence of vessel injury were contributing factors to primary/secondary amputation or salvage. Factors significantly contributing to primary amputation were age, the need for intubation, the need for ICU care, Gustilo type, the existence of infection at admission, more than 5 cm bone loss, the use of an external fixator, and the presence of vessel injury (Table 3). These factors including center establishment were thus included in multivariate analysis to identify factors that contributed to primary amputation.

**Table 3:** Factors contributing to lower extremity amputation and salvage by χ2 analysis

	**Primary amputation χ2 (p)**	**Secondary amputation χ2 (p)**	**Finally salvage χ2 (p)**
**Sex**	0.005 (> 0.999)	0.274 (> 0.999)	0.002 (> 0.999)
**Age**^a^	0.001	0.055	0.107
**Height**^a^	0.923	0.291	0.273
**Weight**^a^	0.809	0.274	0.871
**BMI**^a^	0.427	0.693	0.206
**DM**	1.302 (0.338)	0.026 (0.698)	0.214 (0.811)
**HTN**	2.437 (0.122)	2.189 (0.167)	0.405 (0.548)
**Intubation**	14.465 (0.001)	0.754 (0.483)	13.087 (< 0.001)
**Transfusion**	0.192 (0.667)	0.141 (0.707)	0.190 (0.738)
**E-fast**	3.313 (0.097)	0.000 (> 0.999)	0.913 (0.339)
**ICU care**	8.361 (0.004)	2.925 (0.087)	8.692 (0.003)
**Gustilo**‒**Anderson type**	30.916 (< 0.001)	10.818 (0.025)	38.985 (< 0.001)
**Existence of infection**	11.491 (0.002)	0.055 (0.733)	0.804 (0.370)
**Time to emergency operation**^a^	0.442	0.140	0.005
**Bony loss**	38.704 (< 0.001)	2.459 (0.136)	27.743 (< 0.001)
**External fixator**	8.661 (0.003)	3.012 (0.103)	2.346 (0.126)
**Vessel injury**	19.759 (0.001)	28.068 (< 0.001)	33.823 (0.000)
**Angioplasty**	.562 (> 0.999)	1.929 (0.255)	0.004 (> 0.999)
**Plastic department intervention**	1.952 (0.194)	0.144 (0.781)	0.000 (> 0.999)
**Reconstructive option**	4.933 (0.115)	5.574 (0.109)	2.095 (0.569)
**Complication**	2.521 (0.244)	1.370 (0.616)	1.231 (0.390)
**Patient survival**	4.962 (0.082)	0.306 (> 0.999)	5.457 (0.051)

^a^for continuous variables, analyzed with Student’s *t*-test, values represents *t* *p* value

Center establishment was a statistically significant factor in reducing the amputation rate by 0.74-fold (odds ratio 0.74, *p* = 0.026). For each 1-year increase in age, the primary amputation rate increased by 1.05-fold. When initial infection was present, the amputation rate increased by 3.44-fold. Application of an external fixator increased the amputation rate 4.10-fold (Table 4). Similarly, establishment of the trauma center significantly increased the lower limb salvage rate by 1.32-fold (odds ratio 1.32, *p* = 0.007). The need for ICU care decrease the likelihood of limb salvage. Gustilo‒Anderson type IIIB decreased the salvage rate by 0.89-fold. In addition, for each 1-h delay in surgery, the likelihood of final limb salvage decreased by 0.7 times (Table 5).

**Table 4:** Clinical factors related to lower extremity primary amputation based on multivariate analysis

		**Odds ratio**	**95% CI**		***p*-value**
**Group 1 vs 2**		0.739	0.22	0.862	0.026
**Age**		1.048	1.01	1.088	0.013
**Cause of injury**	**Out car TA**				0.685
	**In car TA**	0.945	0.185	4.833	0.946
	**Fall down**	0.000	0.000	-	0.997
	**Contusion**	0.104	0.005	2.005	0.134
	**Motorcycle**	0.771	0.148	4.018	0.758
**Intubation**		1.061	0.234	4.818	0.939
**ICU care**		2.762	0.504	15.124	0.242
**Gustilo type**	**L**				0.262
	**Ll**	1.17	0.083	16.581	0.908
	**lllA**	1.207	0.06	24.375	0.903
	**lllB**	5.513	0.356	85.441	0.222
	**lllC**	7.09	0.684	73.522	0.101
**Existence of infection**		3.44	1.013	11.677	0.048
**Bony loss**	** < 5 cm**				0.691
	** ≥ 5 cm**	5.303	0.881	31.902	0.068
**External fixator**		4.169	1.353	49.262	0.002
**Vessel injury**	**ATA**	3.183	0.349	29.047	0.305
	**PTA**	0.409	0.021	7.809	0.552
	**Peroneal**	0.000	0.000	-	> 0.999
	**Others**	5.618	0.271	116.542	0.265

*ATA* Anterior tibial artery, *PTA* Posterior tibial artery

**Table 5:** Multivariate analysis of clinical factors related to lower extremity final salvage

		Odds ratio	95% CI		*p*-value
Group 1 vs 2		1.322	1.161	2.267	0.007
Intubation		1.915	0.861	4.26	0.111
ICU care		0.487	0.201	0.081	0.011
Gustilo‒Anderson type	l				0.013
	ll	0.789	0.192	3.251	0.743
	lllA	0.695	0.12	4.017	0.685
	lllB	0.89	0.018	0.957	0.004
	lllC	0.257	0.064	1.03	0.055
Time to emergency operation		0.739	0.201	0.972	0.006
Bony loss	< 5 cm				0.420
	≥ 5 cm	0.521	0.159	1.713	0.283
Vessel injury	ATA	0.562	0.115	2.747	0.476
	PTA	0.246	0.045	1.356	0.107
	Peroneal	0.000	0.000		> 0.999
	Others	0.296	0.044	1.995	0.211

*ATA* Anterior tibial artery, *PTA* Posterior tibial artery

## Discussion

In Korea, social interest in trauma treatment was increased due to a specific event in which a civilian suffered a gunshot injury after being kidnapped by Somali pirates. This highlighted the importance of a trauma management system in Korea. With this increased social demand, the government supported the establishment of Level-1 trauma centers in tertiary hospitals across the country. A Level-1 trauma center is a comprehensive regional resource, comprising a tertiary care facility central to the trauma system. Such centers is capable of providing total care for every aspect of traumatic injury, from prevention through rehabilitation. Following several years of development of a trauma center at our institution, a Level-1 trauma center was established in 2016.

Prior to the establishment of level-1 trauma center, trauma patients were treated through the emergency room. In Trauma-bay, which is part of the emergency room, after the initial treatment by the emergency medicine doctor, the trauma team's activation led to the trauma surgeon's intervention. After the level-1 trauma center was established, Trauma-bay, an independent initial response space like an independent institution, was equipped with independent CT, angiography facility, etc., and also 3 Trauma intensive care units, 3 Trauma operation rooms, and an independent wards and treated patients under a standardized and independent system. Staffing was also added, with orthopedic surgeons, neurosurgeons, radiologists, anesthesiologists, and specialized nursing personnel and coordinators being placed around the trauma surgeon to perform organic and prompt roles. These changes appear to have been the driving force behind the positive changes seen in this study.

Management of lower extremity trauma patients in our institution focuses on the emergency department, but the cooperation of specialists from supporting departments is also essential in open lower extremity trauma. The time to the intervention from the time of the patient’s arrival at the trauma bay is crucial. Early radical debridement, skeletal fixation, and soft tissue coverage has long been known to reduce complications, such as osteomyelitis and nonunion [14]. There are reports that early reconstruction, within 72 h of injury, may lead to lower flap failure rates, lower infection rates, shorter bone healing times, shorter hospital stays, and fewer overall operative procedures [15, 16]. In actual clinical practice, establishing a consensus on the reconstructive intervention timing between physicians from different departments is problematic. However, in our newly established trauma center system, achieving this consensus is easy, and this is directly linked to our higher lower extremity salvage rates. In our study, in patients requiring amputation, only 30% of cases required plastic surgery services in the injury evaluation. Moreover, our system facilitated active inter-departmental transfer of patients according to intensive care team decision. Most of the cases are initially treated by physicians from the trauma surgery department, usually with the patient in the ICU. Then, physicians in the orthopedic department perform surgery for bone fixation, and concurrently soft tissue defects are managed by the plastic surgery department. Regular wound follow-up is performed by the plastic surgeon, and regular wound meetings are held with all involved departments. In this way, physicians can share the burdens of a long treatment period.

In respect to reconstruction of lower extremity trauma, we have observed a marked paradigm shift occurring with the establishment of your trauma center. Advances in microsurgery have made it possible to reconstruct a fairly large soft tissue defect by using the vascularized free tissue transfer technique [17, 18]. For instance, vascularized free tissue transfer has become indispensable Gustilo‒Anderson type IIIB/IIIC open fractures in the trauma setting. Thus, limbs with traumatic injury involving extensive soft tissue loss that in the past would have required amputation can now be preserved because of the developments in microsurgery. These advances have improved the limb salvage rate markedly, and have highlighted the role of plastic surgeons in treating lower extremity trauma at trauma centers. Nevertheless, amputation remains a valid option for severe open fractures with soft tissue defects.

Limb salvage does not necessarily improve the patient's quality of life, but the existence of amputation was evaluated as benefiting because it has an important value in Korea's cultural and spiritual aspects. Approaching and analyzing the quality of life of major trauma patients through long-term follow-up through level-1 trauma center operation is also considered to be an important topic for further research.

Our experience has highlighted three marked changes that occurred with the establishment of a Level-1 trauma center in terms of management of lower extremity traumatic injuries. First, proportion of patients open lower extremity traumatic injuries who have existing infection at their initial visit to our institution was increased after establishment of the trauma center. In contrast the proportion of patients with initial application of an external fixator was decreased. The application of an external fixator usually leads to delayed reduction/fixation and soft tissue coverage. The increased proportion of cases with infected wounds indicated that the severity of the open wound injuries had increased due to regional trauma center system, although use of an external fixator, linked to early reduction, had decreased. Reduced use of an external fixator facilitates early soft tissue coverage, which is key to our successful outcome. Second, we observed a marked reduction in the interval to emergency surgery (on average 2.24 h), which is directly due to establishment of an outstanding trauma center. To ensure early operation, it is necessary that all staff required, including internal medicine specialists, anesthesiologists, and radiologists, are available as like as independent institute. Integration of these services department makes early bone reduction and other essential vascular intervention possible. Third, active plastic surgeon intervention allows reconstruction that directly contributes to limb salvage. The main trauma surgeons cooperate with the reconstructive surgeon in the wound management, and both are actively involved in the overall patient treatment.

In this study, the primary amputation rate significantly decreased and the lower limb salvage rate significantly increased. Among other factors, our analysis showed that establishment of the center contributed significantly to these outcomes. Rapid emergency surgery contributes to limb salvage. However, other clinical factors, such as age, the need for ICU care, and requirement for intubation, which reflect patients’ severity, should also be considered. Additionally, we have demonstrated that the proportion of patients with severe lower extremity trauma patients, such as those classified as Gustilo‒Anderson type IIIB/IIIC, has increased, and the salvage rate has decreased. This finding correlated with those of recent reports that the rate of secondary amputation after revascularization of open tibial fractures was in the range of 10.2‒19.4% [19, 20]. By comparing data from before and after the opening of our trauma center, it was clear that establishment of the trauma center had increased the limb salvage rate; however, severe open fracture cases inevitably suffered limb loss. The increase in our limb salvage rate is encouraging. Thus, orthoplastic surgery needs to be developed further, procedures must continue to improve, and further prospective studies should be performed to assess the outcomes of reconstruction programs.

## Conclusion

With the establishment of our Level-1 trauma center, limb salvage was possible in more patients with open lower extremity fractures, and the rate of primary amputation decreased. Although more severe trauma cases were seen after the center’s establishment, the existence of the center ensured the availability of sufficient and appropriate personnel. Early control of initial open wound infection and minimizing external fixator use allowed early soft tissue reconstruction. A reduced delay to emergency surgery under a well-organized center and with interdepartmental cooperation facilitated active limb salvage, which contributes to patients’ quality of life.

## Data Availability

Possible when it requested. Contact to minjikim1031@naver.com.
